# Society Proceedings

**Published:** 1890-02

**Authors:** W. H. Ridge

**Affiliations:** Secretary


### SOCIETY PROCEEDINGS.

*The Keystone Veterinary Medical Association* held its monthly meeting at the Veterinary Department, University of Pennsylvania, January 4th, 1890. Dr. Huidekoper being absent, Dr. Hoskins, vice-president, presided. At roll call eleven members responded. The minutes of the December meeting were read and approved. The Legislative Committee had nothing new to report. Under unfinished business the change of place of meeting to a more central locality was taken up and discussed by Drs. Zuill, Kooker and Hoskins. The Secretary read a letter from Dr. Marshall, dean of Veterinary Department, stating "that he would be glad to have the Society hold its meetings at the Veterinary Department," but owing to the large number of members that are inconvenienced by the distance the motion was carried to have the meetings in the future in a more central locality. The Secretary was instructed to thank Dr. Marshall for his kind invitation to continue our meetings at the Veterinary Department.

The Secretary read a communication from the Long Island Association, asking the Keystone to endorse their Army Veterinary bill, which was ordered to be laid on the table.

Dr. Zuill moved that Drs. Bachman and Mattson be elected active members of the Association, which was carried. Dr. Kooker moved that a committee of two be appointed to find a place of meeting and also to have power to act. Drs. Kooker and Magill were appointed. There was a resolution offered to amend the by-laws, as follows: Amendment 1: "Members who cannot comply by reading a paper, at their appointed month, can, by notifying the Secretary one month previous, have another member substituted to fill the vacancy." Alteration to Sec. 4, Art. IV., making it read: "Each member shall be entitled to two months' notice for preparation," signed by Drs. Kooker and Ridge. Also resolution to strike out all by-laws relating to associate members and expunge the same from the by-laws, signed by Drs. Zuill and Hoskins. Dr. Ridge read a paper on Parturient Apoplexy. Dr. Zuill exhibited a kidney that had a mulberry calculus in it, also a cyclocephalian monstrosity; subject, a foal. Dr. Eves reported a case of laminitis complicated with tetanus.

W. H. RIDGE, *Secretary*.

